# Identification of a Novel Isolate of the Poultry Roundworm *Ascaridia galli* and Its Egg Embryo Developmental Dynamics In Vitro

**DOI:** 10.3390/vetsci13080827

**Published:** 2026-08-19

**Authors:** Zhaoruiyi Li, Xuan Zhou, Yuxin Qin, Lidan Wang, Xinhui Zhang, Sara Guesmia, Yue Xie

**Affiliations:** 1 Department of Parasitology, College of Veterinary Medicine, Sichuan Agricultural University, Chengdu 611130, China; tango23lz@163.com (Z.L.); 13356645735@163.com (Y.Q.); 2024103034@stu.sicau.edu.cn (L.W.); zzzhangxinhui@163.com (X.Z.); saraguesmia3@gmail.com (S.G.); 2 College of Chemistry and Life Science, Chengdu Normal University, Chengdu 611130, China; zhouxuan198866@163.com; 3 Agricultural Animal Diseases and Veterinary Public Health Key Laboratory of Sichuan Province, Sichuan Agricultural University, Chengdu 611130, China

**Keywords:** avian ascaridiasis, morphology, mitogenomics, embryo development, egg stages

## Abstract

*Ascaridia galli* is a major gastrointestinal nematode of poultry that causes significant economic losses worldwide. In this study, we characterized a new isolate, *A. galli* AgSC_2025, obtained from Xichuan black-bone chickens in China, using an integrative approach by combining morphology, scanning electron microscopy (SEM) and mitogenomics and then its complete developmental changes in eggs—the primary source of infection—was also documented using a high-resolution auto-microscopic imaging. The adult worms exhibited diagnostic characteristics of *A. galli*, including three distinct lips and a specific arrangement of male caudal papillae. Molecular analysis showed a 99.84% mitogenome identity with previously reported *A. galli* and positioned the isolate and *A. galli* in one branch which was sister to other congeneric species within the *Ascaridia* clade. Furthermore, the high-resolution auto-microscopic imaging system yielded a detailed and complete in vitro egg developmental dynamics with thirteen distinct stages, ranging from the initial 1-cell stage to the infective third-stage larva (L3); and further developmental timeline emphasized that the infective L3 larvae predominated by day 10, with 100% reaching this stage on day 13. Viability assays demonstrated a 79.8% ± 0.46% larval hatching rate and the high vitality of the liberated larvae among these infective eggs. These findings provide a comprehensive morphological and molecular reference for this *A. galli* isolate and also establish precise developmental biological parameters for egg embryonation, which should offer valuable insights for species-specific identification and the optimization of sanitation protocols to control avian ascaridiasis.

## Simple Summary

*Ascaridia galli* is a common intestinal roundworm that infects poultry, causing serious health issues and financial losses for the global poultry industry. In this study, we identified and characterized a new roundworm strain isolated from indigenous Xichuan black-bone chickens in China. Using advanced imaging and genetic sequencing, we confirmed its species identity and mapped its genetic makeup. Then, we tracked its egg development in real time using a high-resolution automated imaging system. *A. galli* eggs developed through 13 distinct stages, fully maturing into infective larvae within 13 days, with nearly 80% successfully hatching into active, highly viable larvae. Such high-resolution stage divisions of egg embryonation and a precise chronological timeline to the infective stage fill a critical knowledge gap regarding the early life cycle of this major poultry pathogen and will offer a robust framework for veterinarians and farmers to optimize the control strategies against ascaridiasis in poultry.

## 1. Introduction

The roundworm *Ascaridia galli* (Nematoda: Ascaridida) is one of the most prevalent gastrointestinal nematodes infecting chickens and causes significant adverse effects on the poultry industry through reduced nutrient absorption and growth retardation and decreased egg production [1,2,3,4,5]. Increased epidemiological evidence has highlighted the global burden of this roundworm, with frequent prevalence rates reported in developed countries such as the UK, Germany, and Italy and high prevalence rates (15.6–61%) in developing countries including Ghana, Serbia, Tanzania, Bangladesh, India, and China [6,7,8,9]. Infection with *A. galli* is particularly prevalent in organic or free-range poultry systems, in which the chickens become infected by ingestion of eggs from the contaminated soil and water [1,8]. Following ingestion, the infective third-stage larvae (L3) are released from eggs and initiate the histotrophic phase in the mucosa of the small intestine, and then these larvae reappear in the intestinal lumen and mature into adults [10,11]. Infected chickens become anemic and suffer from diarrhea [12,13].

Clinically, infections with *A. galli* are diagnosed on the basis of the presence of eggs or adult worms in chicken feces, yet such morphology-based identification is often limited by the phenotypic plasticity and close resemblance among congeneric species, such as *Ascaridia columbae* and *Ascaridia dissimilis* [14]. To address this limitation, molecular methodologies, particularly genetic analysis using mitochondrial DNA (mtDNA), have proven to be powerful tools for precise taxonomic resolution in Ascaridida due to its fast mutation rate, low level of recombination and maternal inheritance [15,16]. Importantly, the complete mitogenome data can yield insights into the phylomitogenomic-based species characterization and therefore is being widely used for species-specific identification and differentiation among ascaridoid parasites [15,17]. For example, Liu et al. [18] sequenced the mitogenomes of three ascaridoid species of birds and redefined their taxonomic relationships within Ascaridomorpha. Han et al. [19] assembled the mitogenomes of ten ascaridoid species to characterize their classification status and determined the divergence time of both Ascaridoidea and Heterakoidea families at the early Carboniferous Period. Moreover, Chen et al. [20] described *Hysterothylacium hainanense* as a new roundworm species using integrative morphological and mitochondrial evidence.

In addition, as the primary source of infection in poultry flocks, the embryonated egg is the critical control point for *A. galli* [21]. Environmental factors, including temperature, humidity, and oxygen availability, all can influence the embryonation rate and the survival of eggs outside the host [22]. Theoretically, these eggs embryonate and harbor infective third-stage larvae (L3) in two weeks [8] but remain viable for years under optimal conditions [23]. Several techniques have been described to determine the viability of *A. galli* eggs, including morphological criteria, incubation and flotation techniques, use of dyes, larval motility induction, and animal infection models [22,24]. None of these methods consider a developing embryo—prior to larval stages—as potentially viable. Importantly, information on the early developmental stages and the time at which stage-specific morphological changes occur, as well as the speed of development of *A. galli*, from fertilized to the infective stage, is still lacking. Correctly identifying stages of development is essential for differentiating viable and nonviable parasite eggs and therefore can be used for assessing environmental contamination risks and optimizing sanitation protocols, such as the timing of litter removal or composting in poultry flocks.

The main objectives of this study were to describe a new isolate, *A. galli* AgSC_2025, obtained from a Xichuan black-bone chicken breeding farm, Sichuan Province, China using a combination of morphological and mitogenome-based molecular data and to document the complete in vitro developmental changes in eggs of this isolate using a high-resolution auto-microscopic imaging system. These morphological, mitogenomic, and embryonic development parameters would refine the taxonomic and developmental baseline of *A. galli* lineages and offer a robust framework for veterinarians and farmers to optimize strategies for the prevention and control of chicken ascaridiasis.

## 2. Materials and Methods

### 2.1. Parasites

In August 2025, 120 fresh roundworm specimens were collected from the small intestines of ten Xichuan black-bone chickens (routine autopsy) raised in a breeding farm in Anyue county, Sichuan, China. After thorough washing in sterile physiological saline, worms were transferred into 200 mL glass jars containing RPMI medium (Sigma-Aldrich, St. Louis, MO, USA) supplemented with penicillin (200 μg/mL), streptomycin (200 U/mL) and amphotericin B (10 μg/mL) for 2 days in vitro culture at 37 °C [22,25]. The medium was replaced every 24 h and parasite eggs were collected by centrifugation at 425× *g* for 1 min [22], quantified using a modified McMaster method as described by Feyera et al. [22] and Shifaw et al. [26], and stored in sterile water at 4 °C until use.

### 2.2. Morphological Examinations

All specimens were identified based on macro- and micro-morphological characteristics. Preliminary examination was performed under a stereoscopic microscope (Olympus Corporation, Tokyo, Japan). Body length and width, esophageal dimensions, tail length, and the distance from the nerve ring to the anterior end were measured (mm) using a calibrated micrometer; the egg size was also recorded (µm). Ten representative specimens (five per gender) were further examined by scanning electron microscopy (SEM; Hitachi SU70, Hitachi Ltd., Tokyo, Japan) as described by Álvarez-Fernández et al. [27] (2.5% glutaraldehyde fixation, graded ethanol dehydration, critical-point drying, gold-palladium sputter coating, and SEM imaging at 10 kV).

### 2.3. Mitogenome-Based Phylogenetics

Following morphological examinations, one representative specimen was chosen for molecular identification. High-quality genomic DNA (gDNA) was extracted using TIANamp Genomic DNA kit (TIANGEN, Beijing, China). Briefly, the worm sample was homogenized and digested with Buffer GA and Proteinase K at 56 °C until completely lysed. After incubation with Buffer GB at 70 °C for 10 min and addition of ethanol, the lysate was transferred into a CB3 spin column, washed with Buffers GD and PW, and eluted with Buffer TE. And its concentration and purity were assessed using a NanoDrop spectrophotometer (Thermo Fisher Scientific, Waltham, MA, USA), while its integrity was checked by 1.0% agarose gel electrophoresis. The gDNA was used to construct a paired-end library and sequenced on an Illumina NovaSeq platform (150 bp paired-end reads). Raw reads were quality-filtered (adapter/poly-N/low-quality reads; Q ≤ 20) using fastp software v0.23.4 [28] and were de novo assembled into the complete mitogenome using IDBA-UD [29]. The genome was annotated using MITOS [30] and manually checked by alignment in Geneious v2025.1.3. The genome identity was compared against available Ascaridida mitogenomes using mVISTA and phylogenetic inferences were performed using Maximum Parsimony (MP), Maximum Likelihood (ML) using IQ-TREE v2.2.0 [31], and Bayesian Inference (BI) using MrBayes v3.2.7a [32] to confirm species identity in Ascaridida [33]. For both ML and BI analyses, the GTR + I + G nucleotide substitution model was utilized. Nodal support was evaluated with 1000 bootstrap replicates for the ML tree. For the BI analysis, Markov chain Monte Carlo (MCMC) simulations were run for 10,000,000 generations, with sampling performed every 1000 generations. Based on combined morphological and molecular evidence, the isolate from Xichuan black-bone chickens in Anyue of Sichuan (China) was identified as a new isolate of *A. galli* (*A. galli* AgSC_2025).

### 2.4. Egg Developmental Dynamics of A. galli AgSC_2025 In Vitro

Embryo development of *A. galli* AgSC_2025 was profiled over 21 days in vitro using a high-resolution automated imaging system (ICX41 plus, Sunny Optical Technology [Group] Company Limited, Ningbo, China). Specifically, eggs were incubated at approximately 200 eggs/mL in RPMI containing penicillin (200 μg/mL), streptomycin (200 U/mL) and amphotericin B (10 μg/mL) [25] at 28 °C, as described previously [34,35,36]. One-milliliter aliquots were transferred to McMaster chambers as observation vessels and the volume was maintained daily by adding fresh, oxygenated RPMI medium against evaporation. Embryo morphology was photographed and recorded every 12 h. Developmental stage division and stage proportion were defined as described previously [37,38]. At the end of incubation, the egg viability was assessed by in vitro larval hatching using a modified method: eggs were treated with 4% NaOH and 4% NaClO overnight, incubated in 0.2% Tween-80 for 60 min, washed in PBS three times, and centrifuged at 930× *g* for 10 min to promote hatching. Eggs and hatched larvae were stained with methylene blue (1:10,000) and the viability was calculated as the proportion of methylene blue-unstained larvae [39,40].

### 2.5. Statistical Analysis

The developmental stage percentages were expressed as the proportion of eggs assigned to each stage at each observation time point. Stage proportions were calculated with triplicate counts of tested eggs and the egg viability was calculated with triplicate counts of unstained larvae over tested eggs. All data were tabulated in Microsoft Excel and the developmental dynamics and chronological progression were visualized using GraphPad Prism v9.0.

## 3. Results

### 3.1. Identification of a New Isolate of A. galli AgSC_2025

#### 3.1.1. Morphological Characteristics

**Figure 1:**
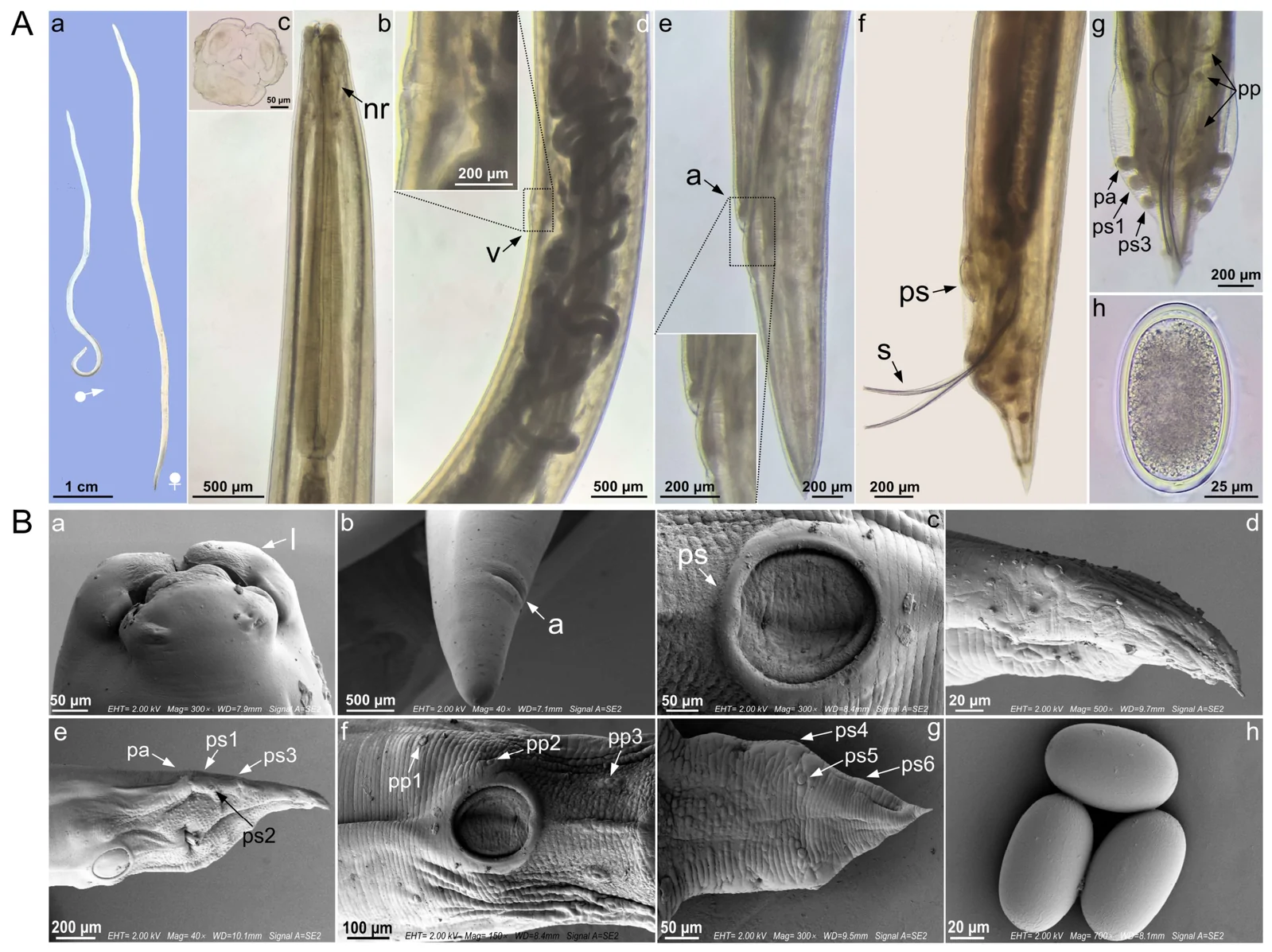
Morphological characteristics of *Ascaridia galli*. (**A**) Gross morphology and light microscopic observations. (**a**) Macroscopic view of adult worms, showing a male specimen on the left and a female specimen on the right. (**b**) Anterior end, showing the nerve ring. (**c**) Three trilobed lips. (**d**) Vulva region of the female worm, showing the vulva. (**e**) Lateral view of the posterior end of the female worm, showing the anus. (**f**) Lateral view of the posterior end of the male worm, showing the spicule and precloacal sucker. (**g**) Ventral view of the posterior end of the male worm, showing the precloacal sucker and caudal papillae. (**h**) Egg. (**B**) Scanning electron microscopic observations. (**a**) Dorsal view of the anterior end, showing the three trilobed lips. (**b**) Posterior end of the female worm, showing the anus. (**c**) Ventral view of the male precloacal sucker. (**d**) Lateral view of the tail tip of the male worm. (**e**) Lateral view of the posterior end of the male worm, showing the precloacal sucker and caudal papillae. (**f**) Region of the precloacal sucker, showing the precloacal papillae. (**g**) Ventral view of the tail tip of the male worm, showing the 4th–6th postcloacal papillae. (**h**) Eggs. Abbreviations: nr—nerve ring, v—vulva, a—anus, ps—precloacal sucker, s—spicule, l—lip, pp—precloacal papillae, pp1–3—1st–3rd precloacal papillae, pa—paracloacal papillae, and ps1–6—1st–6th postcloacal papillae.

Morphologically, adult worms were cylindrical and semitransparent milky-white in appearance. Sexual dimorphism was distinct, with females (63.81–80.56 mm) longer than males (35.64–61.27 mm), and the male tail was coiled while the posterior end of the female was straight and pointed (Figure 1(Aa); Table 1). Under light microscopy, the entire body was covered with transversally striated cuticle, and the mouth at the anterior end of the adults was surrounded by three lips with nearly equal size, esophagus club-shaped without a distal bulb (Figure 1(Ab,Ac)). The male tail was slender and pointed, curving slightly ventrally with a pair of equal-length spicules extended from the cloaca, and a circular precloacal sucker, unique to males, was located anterior to the cloaca with three pairs of precloacal papillae (Figure 1(Af,Ag)). In females, the vulva was situated in the mid-body region; it appeared smooth and lacked papillae. The relatively long vagina extended anteriorly to connect with the undivided portion of the uterus, and the anus was visible at the posterior end (Figure 1(Ad,Ae)). The size of the egg was measured as 62–89 µm × 43–51 µm (Figure 1(Ah)). Such characteristics were identical with the descriptions of *A. galli* [41]. Micro-morphological characteristics under SEM further confirmed the species identity (Figure 1B). It was clear that three distinct lips featured pronounced, paired fleshy protrusions with their length greater than width (Figure 1(Ba)). The female had a blunt and rounded posterior end with a single large and open anus before the tip of its tail (Figure 1(Bb)). In contrast, the male had a comparatively elaborated and more complex posterior end: an oval precloacal sucker with its longitudinal diameter greater than transverse diameter, and this sucker rim was well-developed and its spherical ridge formed a sclerotized ring with an elliptical protrusion at the center (Figure 1(Bc)). Additionally, the extreme terminal tip was pointed and slightly expanded at the base (Figure 1(Bd)). Notably, the male tail also had ten papillae on each side: three on a precloacal position, one on an adanal position, and six on a postanal position (Figure 1(Be–Bg)). The eggs were oval, with a smooth eggshell surface (Figure 1(Bh)).

#### 3.1.2. Molecular Characteristics

**Figure 2:**
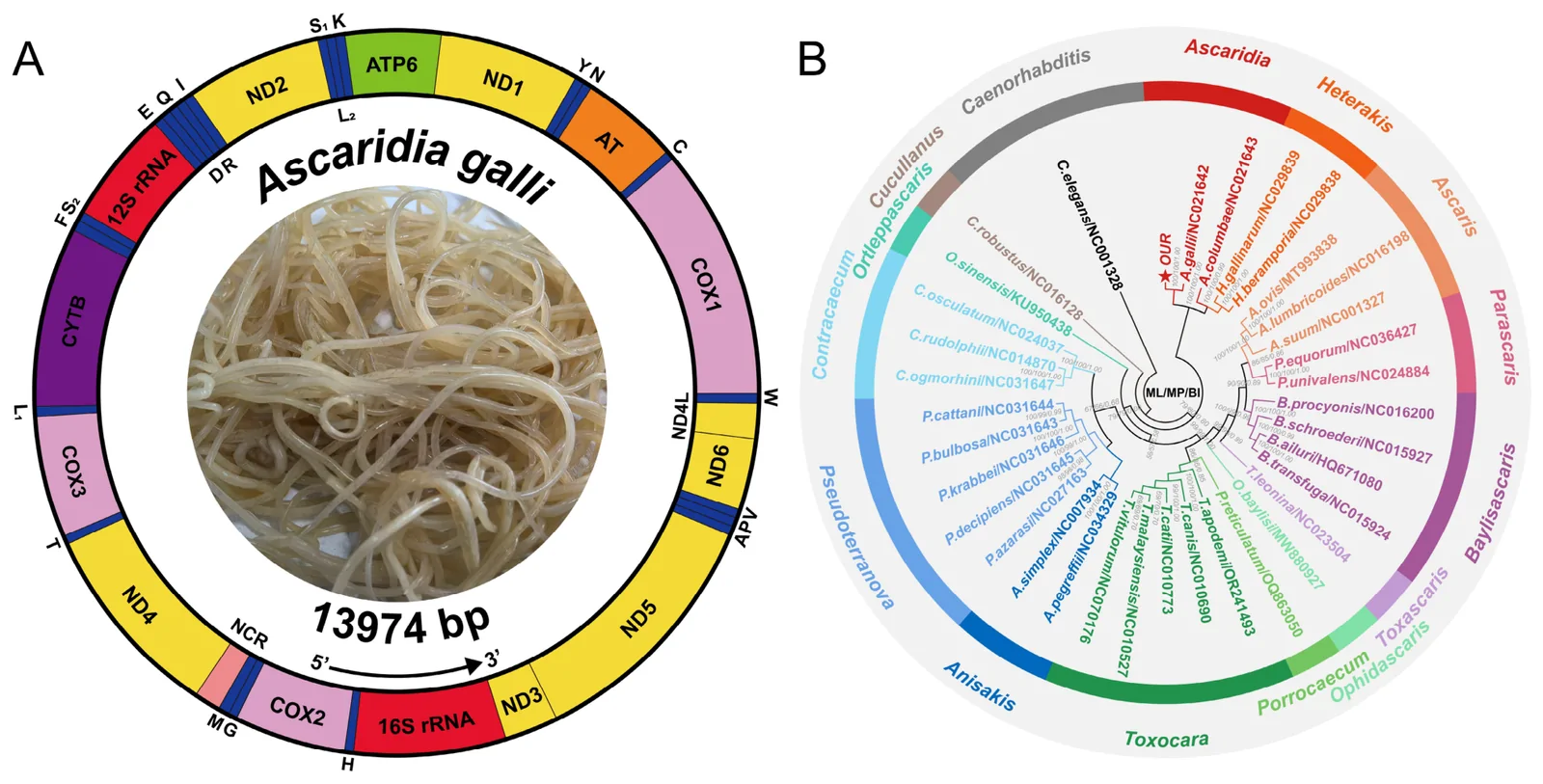
Mitochondrial genome and phylogenetic relationships of *Ascaridia galli*. (**A**) Circular representation of the *A. galli* mitogenome. All genes are on the same DNA strand and are transcribed anticlockwise. Twenty-two tRNAs are represented by single letters corresponding to their respective amino acid codes. Two leucine genes are distinguished as L1 and L2, and two serine genes are labeled as S1 and S2. NCR denotes the non-coding region, and AT denotes the AT-rich region. (**B**) Phylogenetic tree of *A. galli* and related nematodes reconstructed based on the nucleotide sequences of 12 concatenated protein-coding genes using Maximum Likelihood (ML), Maximum Parsimony (MP), and Bayesian Inference (BI) methods. *Caenorhabditis elegans* was used as the outgroup. The red asterisk represents the mitochondrial genome of *A. galli* sequenced in this study. Numbers along the branches indicate nodal support values in the order ML/MP/BI. Bootstrap values lower than 50 and Bayesian posterior probabilities lower than 0.50 are not shown.

In parallel with morphological identifications, our molecular identification revealed a 13,974 bp complete mitogenome (GenBank accession no. PZ559952) harbored in the isolate, which encoded 12 protein-coding genes (PCGs; ATPase subunit 6 [*ATP6*], cytochrome c oxidase subunits 1-3 [*COX1-3*], cytochrome b [*CYTB*], and NADH dehydrogenase subunits 1-6 and 4L [*ND1-6* and *ND4L*]), 22 tRNAs, and 2 rRNAs (Figure 2A) and shared an identical gene arrangement and composition organization to that of the *A. galli* mitogenome reported by Liu et al. [18]. Further, the whole mitogenome sequence identity of this isolate with that of other published Ascaridida nematodes was estimated using mVISTA and the respective genome-wide identities (in decreasing order) were *A. galli* (Hunan isolate; 99.84%), species of *Heterakis* (81.11–81.27%), *Ascaridia columbae* (79.05%), species of *Ascaris* (55.15–55.49%), *Toxascaris_leonina* (55.04%), species of *Parascaris* (54.77–54.94%), species of *Toxocara* (54.19–54.67%), species of *Pseudoterranova* (54.31–54.66%), species of *Contracecum* (54.32–54.58%), species of *Baylisascaris* (54.17–54.55%), species of *Anisakis* (54.13–54.51%), *Porrocaecum reticulatumm* (54.48%), *Ortleppascaris sinensis* (54.40%), *Ophidascaris baylisi* (54.18%), *Caenorhabditis elegans* (53.84%), and *Cucullanus robustus* (53.82%). It was noteworthy that the isolate and *A. galli* genomes exhibited the highest percentage identity of 99.84%. In addition, based on the concatenated amino acid sequences of 12 PCGs, our phylogenetic analyses (MP/ML/BI) consistently positioned the isolate and *A. galli* (NC_021642) as one group in the branch, and both were sister to *Ascaridia columbae* (NC_021643) within *Ascaridia*, with each node having maximum bootstrap support (all values = 100 or =1.00) (Figure 2B). Combined, these genome-wide data supported that this isolate and *A. galli* were a single species, namely, *A. galli* AgSC_2025.

### 3.2. Embryo Developmental Dynamics of A. galli AgSC_2025 Eggs

#### 3.2.1. Developmental Stage Divisions and Morphological Changes

**Figure 3:**
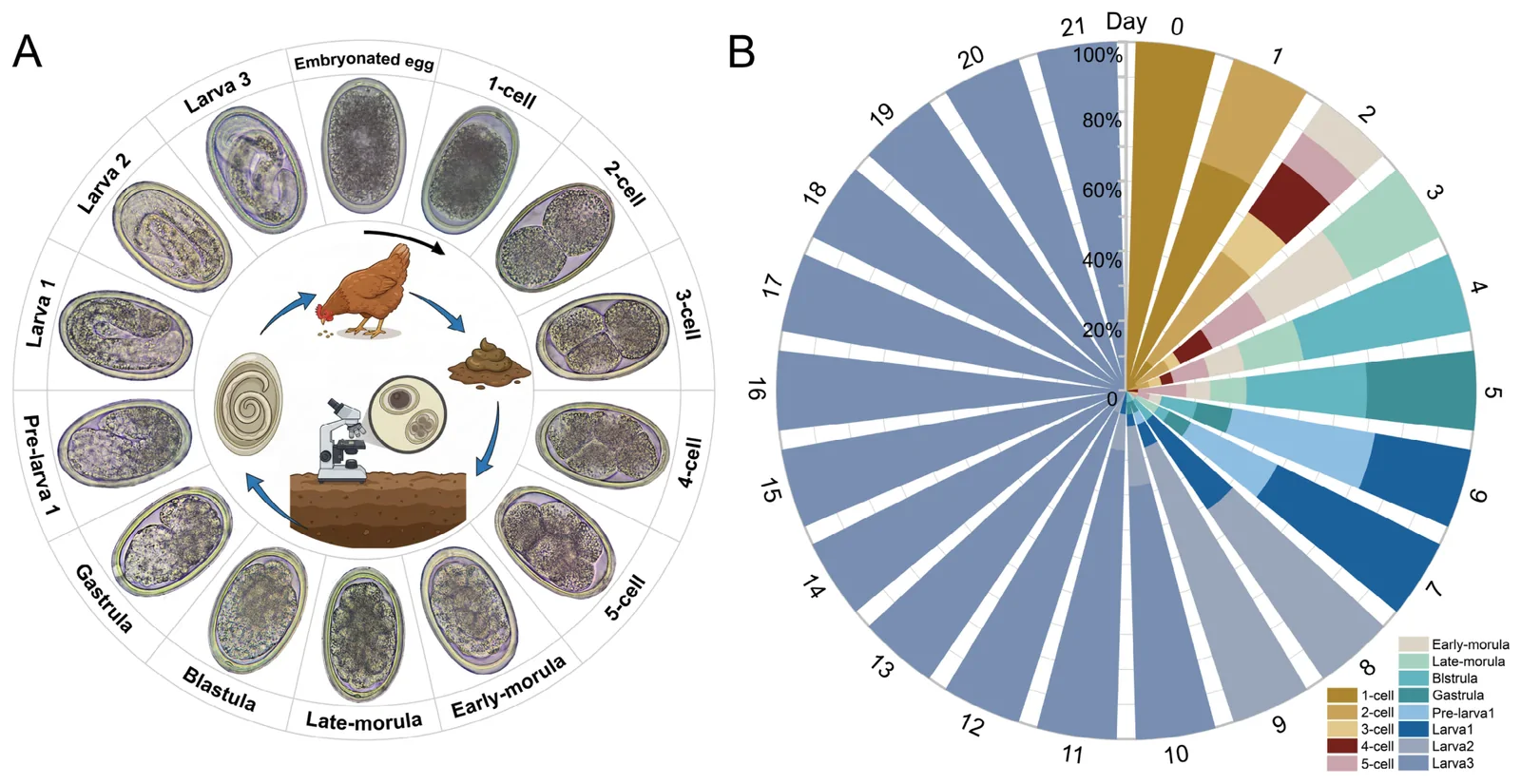
Developmental dynamics of *Ascaridia galli* eggs. (**A**) Schematic representation of the developmental trajectory of *A. galli*. The outer circle illustrates the sequential morphological progression of eggs, with black arrows indicating the direction of egg development. The inner circle depicts the life cycle of *A. galli*, with blue arrows indicating the direction of life-cycle progression. (**B**) Temporal changes in the proportions of different developmental stages during in vitro culture at 28 °C for 21 days.

After the species identification on the basis of combined morphological and molecular evidence, the eggs obtained from the remaining representatives of *A. galli* AgSC_2025 were cultured in McMaster counting chambers for 21 days at 28 °C. During this period, a total of thirteen distinct developmental stages including 1-cell, 2-cell, 3-cell, 4-cell, 5-cell, early-morula, late-morula, blastula, gastrula, pre-L1 (early first-stage larval form), L1 (first-stage larva), L2 (second-stage larva), and L3 (infective stage larva) were recorded and described (Figure 3A). Specifically, fresh eggs of *A. galli* AgSC_2025 were elliptical with a thick shell and were almost completely filled with the cytoplasm. After a few hours of incubation, the cytoplasm of the eggs gradually condensed and created a clear perivitelline space between the blastomere and inner eggshell, which formed a typical and evident 1-cell morphology, namely, 1-cell stage. Within the next 24 h, most of these eggs underwent the first cleavage and entered the 2-cell stage. Subsequently, cell division continued and sequentially entered 3-cell, 4-cell, and 5-cell stages, followed by compaction into early- and late-morulae over 1–2 days. Around day 4, further visible cell divisions were no longer apparent, and the embryos approached the blastula stage which exhibited a lighter-colored, spherical layer of cells surrounding the inner mass and enclosing a fluid-filled cavity. On day 5, the gastrulation became evident: a localized thickening and infolding of the cell layer on one side of the embryo produced a characteristic “kidney-like” indentation, namely, the gastrula stage. Subsequently, the embryos differentiated into larval forms. By day 6, part of the embryos developed into a distinct larval structure with a “U-shaped” or “6-shaped” configuration, corresponding to the pre-L1 and L1 stages. With the further development of the embryos, the larvae elongated and occupied most of the space of the egg. By day 8, the L2 larvae were clearly visible in the eggs, and they consistently showed an “8-shaped” flexing movement when stimulated by light. From day 10 onwards, the fully developed L3 larvae appeared. Notably, these infective L3 larvae were elongated and coiled within the egg, and exhibited delayed movement in response to light stimulation, typically initiating activity 3–5 min after exposure. No spontaneous hatching of L3 larvae was observed at the end of the 21-day culture period.

#### 3.2.2. Proportional Changes in Various Developmental Stages

It was clear that multiple developmental stages co-occurred at the same time point during our 21-day observations (Figure 3B). Notably, the first 10 days of incubation appeared to represent the main phases of active development, because approximately 10–60% of eggs progressed into a new stage almost every day and the proportion of eggs in that stage peaked on the day it first appeared or on the following day during this period. In addition, the early cleavage stages approached their maximum representation soon after onset. Specifically, on day 2, the proportions of 2-cell, 3-cell, and 4-cell eggs peaked at 38.0%, 13.8%, and 17.2%, respectively. By day 3, 5-cell embryos accounted for 17.2% of the eggs, while early- and late-morula stages each reached their highest proportions at 27.6%. It was noteworthy that the 1-cell stage was last observed on day 3, only accounting for 3.4% of the eggs. Subsequent stages followed a similarly ordered pattern. For example, the blastulae peaked on day 4, comprising 48.3% of the eggs, whereas gastrulae reached their maximum proportion (31.0%) on day 5. On day 6, pre-L1 forms were most abundant, accounting for 41.4% of the eggs. By day 7, the majority of developing embryos progressed to the L1 stage (51.7%). The L2 stage predominated on day 9, and it also reached its peak proportion of 82.8% at this timepoint. The infective L3 stage was observed in large numbers on day 10, and about 72.4% of eggs contained the L3 larvae. By day 13, all embryos developed to the L3 stage, meaning that all eggs became infective.

#### 3.2.3. In Vitro Larval Hatching and Viability Testing

**Figure 4:**
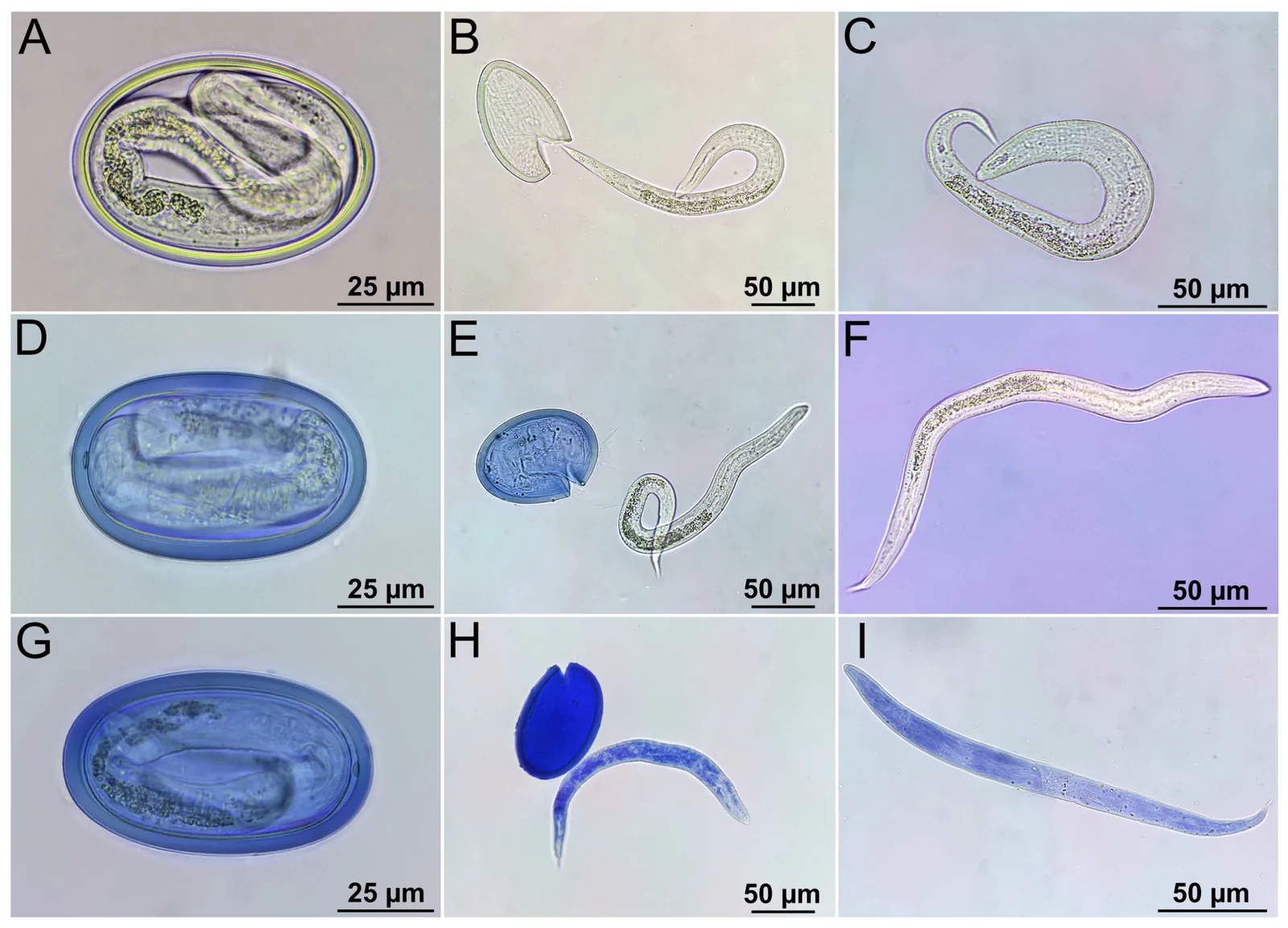
Methylene blue-based viability assessment of *Ascaridia galli* eggs and larvae. (**A**–**C**) Unstained controls showing embryonated eggs and viable larvae. (**D**–**F**) Methylene blue-treated eggs containing viable larvae. The hatched larva remained unstained indicating dye exclusion. (**G**–**I**) Methylene blue-treated eggs containing non-viable larvae. The hatched larva was stained indicating the dead larva absorbed the dye and therefore showed an obvious methylene blue staining.

At the end of the incubation period, 200 embryonated eggs were subjected to in vitro hatching using the modified method by Feyera et al. [22] and the liberated larvae were tested for viability using methylene blue staining. Prior to staining, the viable larvae and embryonated eggs exhibited normal morphology with intact structures (Figure 4A–C). A total larval hatching rate of 79.8% ± 0.46% was determined among the tested eggs. For the remaining unhatched eggs, most of them were stained internally by methylene blue in comparison with the hatched counterparts, suggesting damage to their eggshells and possible larval death. This conclusion was further supported in our viability assessment experiment, in which the hatched larvae remained motile and unstained; by contrast, the larvae of unhatched eggs were non-viable and absorbed the methylene blue (Figure 4D–F vs. Figure 4G–I).

## 4. Discussion

*Ascaridia galli* infection causes severe health problems and significant economic losses in the global poultry industry, necessitating a comprehensive understanding of its de-velopmental biological characteristics, especially those involved in the in vitro developmental dynamics of eggs. In the present study, on the basis of a combination of morphological and mitogenome-based evidence, *A. galli* AgSC_2025 was confirmed as a new isolate, and then its complete egg development was tracked in real-time using a high-resolution auto-microscopic imaging. The high-resolution 13-stage divisions of egg embryonation and a precise chronological timeline to the infective stage filled a critical knowledge gap regarding the early life cycle of *A. galli* and also offered a robust framework for optimization of control strategies against this parasite.

The in vitro morphological progression of *A. galli* eggs documented in the present study complements and expands upon the developmental information previously reported for this species. Although embryonated eggs constitute the primary source of infection for avian ascariasis, previous studies on the in vitro development of *A. galli* eggs have generally focused on limited time points or major developmental categories, and a complete, continuous morphological record has remained insufficient. To address this gap, we continuously cultured and monitored *A. galli* eggs in vitro for 21 days at 28 °C and identified 13 distinct developmental stages, ranging from the 1-cell stage to the L3 stage. Earlier reports [37,46] described the development of *A. galli* eggs using broader staging systems, such as fertile egg, 2-cell, 3-cell, 4-cell, morula, tadpole, vermiform embryo and L3 stages, or fertile egg, cleavage stage, early morula, late morula, blastula, gastrula, larval shape stage and embryonated stage. In comparison, the present study further resolved the early cleavage period by identifying the 1-cell and 5-cell stages in addition to the previously described 2-cell, 3-cell and 4-cell stages, and subdivided the larval transition period into pre-L1, L1, L2 and L3 stages, with L3 representing the infective-stage larva. These findings indicate that the embryonic development of *A. galli* eggs involves a series of gradual and recognizable morphological transitions rather than a simple progression from unembryonated to embryonated eggs. In addition, the present study confirmed the asynchronous nature of *A. galli* egg development under in vitro conditions, a phenomenon that has also been observed in other eggs of ascarid nematodes [47]. Different developmental stages were observed simultaneously within the same culture system; even when some eggs had developed to the infective larval stage, a large proportion of eggs remained at earlier embryonic stages. Such developmental asynchrony may be biologically advantageous, as it may extend the temporal window during which infective eggs are present in the environment, thereby increasing the likelihood of transmission to susceptible chicken hosts. From an epidemiological perspective, this also suggests that environmental contamination with *A. galli* eggs may remain infective over a prolonged period, even when eggs are deposited at the same time.

When compared with other ascaridoid nematodes, the developmental morphology of *A. galli* eggs showed both conserved features and species-specific differences. The 13 developmental stages identified in the present study were highly comparable to those reported for *Toxocara* spp. [48,49,50] and *Ascaris suum* [51], suggesting that the embryonic development of ascaridoid eggs is evolutionarily conserved to some extent. Compared with the twelve developmental stages described for *A. suum* eggs, including the 1-cell, 2-cell, 3-cell, 4-cell, early-morula, late-morula, blastula, gastrula, pre-larva 1, pre-larva2, L1, and L2, the present study delineated two additional stages in *A. galli*, namely the 5-cell and L3 stages. Relative to common *Toxocara* species, the developmental morphology of *A. galli* eggs was largely consistent with that of *T. cati* eggs, although an additional L3 stage was identified in *A. galli* in the present study. However, compared with *T. canis* and *T. vitulorum*, the present study identified two additional stages in *A. galli*, namely the 5-cell and L3 stages. These comparisons indicate that although the general developmental trajectory of ascaridoid eggs is similar, the resolution of staging and the timing of morphological transitions may differ among species. Clear interspecific differences were also observed in developmental rate. In the present study, infective L3 larvae of *A. galli* were first observed on day 10, indicating that *A. galli* eggs developed faster than *A. suum* eggs, which require approximately 16 days to reach the infective stage, but slightly more slowly than *Toxocara* spp., in which infective larvae may appear within 7–9 days. Therefore, the developmental velocity of *A. galli* appears to be intermediate between that of *A. suum* and *Toxocara* spp. We hypothesized that this intermediate rate may be associated with eggshell-related properties. In particular, a thinner and smoother eggshell could facilitate oxygen and water exchange, thereby promoting embryonation and accelerating early development [52]. However, eggshell thickness alone cannot fully explain the observed differences in developmental velocity among ascaridoid nematodes. Other factors, including eggshell permeability, embryonic metabolic activity, energy allocation and species-specific developmental regulation, may also contribute to these differences [53]. The slightly slower development of *A. galli* compared with *Toxocara* spp. may further reflect differences in host ecology and transmission strategies. *Toxocara* spp. parasitize carnivorous or omnivorous mammalian hosts and may exploit complex transmission routes involving environmental eggs, paratenic hosts and predation [54,55,56]. In such transmission systems, rapid embryonation may increase the probability that eggs become infective within short and unpredictable environmental windows. In contrast, *A. galli* is mainly transmitted in poultry houses, litter, soil and free-range environments, where chickens repeatedly peck contaminated substrates and where fecal contamination can accumulate continuously [8,21]. Therefore, *A. galli* may not require the extremely rapid embryonation observed in some *Toxocara* species. Instead, its relatively moderate developmental rate may represent a trade-off between rapid acquisition of infectivity and maintenance of larval viability in frequently contaminated but environmentally fluctuating poultry habitats. Thus, from the perspective of disease prevention and environmental decontamination, these developmental characteristics of *A. galli* eggs underscore the practical necessity to obtain targeted ovicidal agents. Importantly, several studies have demonstrated that chlorocresol-based disinfectants (e.g., Neopredisan^®^ 135-1) have significant inhibition of egg embryonation in *Ascaridia* spp. [37,57]. However, achieving consistent ovicidal efficacy in practical poultry farm environments remains challenging. Factors such as organic load, substrate penetration, and environmental dilution may reduce the disinfectant activity of chlorocresol in real-world settings. Therefore, future research should focus on optimizing the application protocols of chlorocresol-based disinfectants to ensure reliable and reproducible ovicidal effects under field conditions.

However, several limitations should be considered when interpreting the developmental staging system established in this study. First, embryonic changes were recorded at 12 h intervals. Although continuous culturing allowed longitudinal monitoring over 21 days, morphological transitions that occur rapidly between two consecutive observations may have been missed or could have appeared shorter/longer than their actual durations. Therefore, the precise onset timing of specific cleavage divisions may be slightly biased by the temporal resolution. Second, stage assignment was based primarily on morphological criteria observed under light microscopy and guided by previously described staging frameworks [37,38]. During early cleavage and larval transition windows, morphological features can be subtle, and egg-to-egg variability may complicate classification, particularly for eggs captured near the borders between adjacent stages. As a result, a small degree of misclassification cannot be fully excluded. Third, our study reported one novel isolate collected from a single farm. Its developmental rates and consequently morphological traits such as adult body size may vary across geographic strains and environmental conditions. This may help explain the discrepancy in male body length between our measurements (35–61 mm) and Guevara’s report (47–75 mm) based on Peru samples, where the observed male size range differs from that of our China-derived specimens. Fourth, although the complete mitogenome sequencing provided a robust taxonomic resolution for this isolate, using a single representative specimen for genomic analysis represents a limitation. Consequently, the intra-population genetic variation and diversity of this *A. galli* population were not fully assessed in this study. Future studies incorporating a larger cohort of samples from multiple individuals are warranted to thoroughly characterize the population-level genetic diversity and microevolutionary patterns of this parasite.

Methodologically, a McMaster counting chamber was utilized as the culture vessel. This facilitated real-time, longitudinal observation of identical eggs. Moreover, the thin fluid layer within the chamber maximized oxygen contact probability, ensuring normal development and data accuracy. Furthermore, the RPMI culture solution (containing 200 μg/mL penicillin, 200 U/mL streptomycin, and 10 μg/mL amphotericin B) employed in this experiment replaced the traditional 5% formalin solution. This can effectively mitigated the potential health hazards of formalin inhalation for the investigator during frequent observations. Therefore, this optimized protocol warrants further promotion for the in vitro culture of other parasitic helminth eggs and larvae in the laboratory.

## 5. Conclusions

In summary, this study provided an integrative characterization of the *Ascaridia galli* AgSC_2025 isolate through morphological, SEM, and mitogenomic analyses, establishing a robust reference for its taxonomic identification. By utilizing a high-resolution auto-microscopic imaging, we also delineated a total of thirteen distinct egg developmental stages and determined and that 100% infectivity was reached within 13 days, filling a critical gap in the understanding of this parasite’s early life cycle. Meanwhile, the 13-day timeline for egg embryonation also provided a specific window for litter removal to break the transmission cycle of avian ascaridiasis.

## Figures and Tables

**Table 1:** Morphometric comparison of *Ascaridia galli* isolates obtained from various host species and geographic locations (measurements in mm).

Item	This Study		Guevara [42]		Jaiswal et al. [43]		Rao et al. [44]		Zhao et al. [45]	
	Male	Female	Male	Female	Male	Female	Male	Female	Male	Female
BL	35.64–61.27	63.81–80.56	41.0–75.0	67.0–110.0	40–47	40.0–66	42.2–54.2	58.9–74.6	30.0–41.0	39.0–56.0
BW	0.56–0.79	0.98–1.02	0.66–1.29	1.06–2.00	0.40–1.20	1.00–1.63	0.68–0.72	0.93–0.98	-	-
LL	0.11–0.15	0.14–0.17	0.09–0.13	0.13–0.26	-	-	0.12–0.14	0.15–0.18	0.12–0.13	0.13–0.17
LW	0.08–0.10	0.09–0.12	0.10–0.20	0.13–0.33	-	-	0.08–0.09	0.09–0.11	-	-
OL	2.45–4.26	2.36–4.35	2.80–4.50	3.00–5.00	-	-	1.02–1.13	1.03–1.35	1.67–3.26	2.14–3.69
OW	0.30–0.32	0.30–0.42	0.20–0.53	0.26–0.59	-	-	0.28–0.33	0.31–0.39	-	-
NRD	0.23–0.36	0.42–0.48	-	-	-	-	0.21–0.31	0.34–0.39	-	-
PS-L	0.17–0.29	-	0.17–0.30	-	0.20–0.29	-	0.18–0.19	-	0.26–0.31	-
PS-W	0.16–0.20	-	0.17–0.30	-	0.15–0.19	-	0.14–0.16	-	0.15–0.22	-
PSC	0.27–0.45	-	0.26–0.53	-	0.25–0.57	-	0.27–0.28	-	0.27–0.44	-
SL	1.35–2.15	-	0.89–2.57	-	1.65–1.90	-	1.39–1.61	-	1.26–2.43	-
VL	-	27.83–48.69	-	11.0–15.0	-	19–35	-	29.86–38.12	-	19.1–28.6
ES	62–89 × 43–51		67.2–85.2 × 49.2–59.0		-		210–300 × 120–190		-	
Host	*Gallus gallus domesticus*		*Gallus gallus*		*Gallus gallus*		*Gallus gallus domesticus*		*Pavo muticus*	
Distribution	China		Peru		India		China		China	

“-” Mean Measurements were not performed. Abbreviations: BL—length of body, BW—width of body, LL—length of lips, LW—width of lips, OL—length of esophagus, OW—width of oesophagus, NRD—distance from anterior end to nerve ring, PS-L—length of precloacal sucker, PS-W—width of precloacal sucker, PSC—distance from precloacal sucker to cloaca, SL—length of spicule, VL—distance from anterior end to vulva, and ES—size of eggs.

## Data Availability

The original contributions presented in this study are included in the article. Further inquiries can be directed to the corresponding author.
